# The influence of sleep on job satisfaction: examining a serial mediation model of psychological capital and burnout

**DOI:** 10.3389/fpubh.2023.1149367

**Published:** 2023-08-24

**Authors:** Mavis Agyemang Opoku, Seung-Wan Kang, Suk Bong Choi

**Affiliations:** ^1^Gordon S. Lang School of Business and Economics, University of Guelph, Guelph, ON, Canada; ^2^College of Business, Gachon University, Seongnam, Republic of Korea; ^3^College of Global Business, Korea University, Sejong City, Republic of Korea

**Keywords:** sleep, psychological capital, burnout, job satisfaction, serial mediation

## Abstract

**Introduction:**

This study draws on the conservation of resources theory to investigate whether the loss of sleep can trigger the loss of additional resources that are necessary for work.

**Methods:**

Using cross-sectional design of 322 call center employees working at a government-owned public bank in South Korea, we test the study hypotheses using regression and bootstrapping indirect effects analyses.

**Results:**

The results of analyses show that insufficient sleep increases employee burnout and that psychological capital mediates this relationship. We also find that insufficient sleep decreases job satisfaction via a serial mediation model such that insufficient sleep reduces psychological capital, which in turn increases burnout, and ultimately results in lower job satisfaction.

**Discussion:**

The findings reinforce the previous assessment that although sleep is a non-work factor, its impact spills over to the workplace. Theoretically, this study goes beyond direct effect to uncover the underlying or mediating mechanisms that account for the impact of the sleep-burnout relationship and the sleep-job satisfaction relationship. For managers, the results highlight the significance of sleep to employees’ overall health and well-being and thus underscore the need to foster a work culture that recognizes and prioritizes employee sleep needs.

## 1. Introduction

Getting a good night’s sleep is necessary for rejuvenation, performing daily activities, and general well-being. Since the dawn of civilization, scholars in the fields of art, poetry, philosophy, and mythology have been intrigued by the concept of sleep as evidenced in the early works of art, literature, and medicine (1). The 2019 Global Sleep Survey conducted by Phillips revealed that although people recognize the importance of sleep for their overall health, only 10% reported that they sleep extremely well. Another 40% of adults reported that their sleep had worsened over the previous 5 years (2). Consistent with this global trend, studies show a significant decline in the sleep duration of South Korean adults (3). In fact, sleep problems seem to be more pervasive in developed countries (4). For instance, data from a recent Gallup survey indicate that about one in three American adults do not get the recommended amount of sleep (5). In the UK, a third of Britons report getting less than 7 h of sleep per night (6). Similarly, national studies have found a prevalence of sleep problems among Japanese (7) and Swedish adults (8). The global decline in sleep has in part fueled the recent interest in sleep research by organizational scholars, which has provided insight into the effects of sleep within the workplace (9). A meta-analysis on sleep revealed that sleep impacts physical and psychological health, physiological outcomes that affect the ability to process and recognize emotional experiences, attitudinal consequences including organizational commitment and engagement, as well as performance outcomes that include safety behavior, task performance, and contextual performance such as organizational citizenship behavior (9).

Sleep does not merely denote the absence of wakefulness or suspension of the senses but rather it results from physiological processes including the activation of some neurons in certain brain areas and a passive withdrawal of sensorial stimulation from the brain (1). Several factors influence the optimal sleep requirement of each individual. For instance, some researchers have argued that women and older adults may require more sleep time (1) or have difficulty in achieving restorative sleep (10). Prior reviews (11, 12) have identified characteristics of the work environment including workload, work schedules, role conflict, role ambiguity, occupational stressors, interpersonal conflict, perceived control, and situational constraints as organizational antecedents of employees’ sleep time and quality. Accordingly, subjective perceptions of meeting one’s optimum sleep needs have been an important focus for researchers since this may capture the multi-faceted nature of individual sleep needs more comprehensively. This focus is reflected in empirical sleep research where sleep has often been operationalized in terms of the number of hours spent sleeping (sleep quantity) or difficulty in sleeping, staying asleep, or feeling rested after sleep (sleep quality) (9). This study focused on the subjective perceptions of having insufficient sleep time.

**Figure 1:**
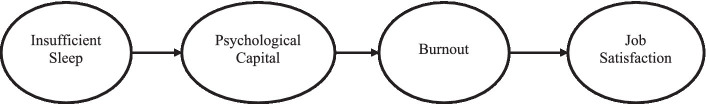
Hypothesized research model.

Given that sleep is an invaluable resource, we use the conservation of resources (COR) (13, 14), a resource-based theory to ground our research model. The basic tenet of the COR theory suggests that people are motivated to retain, build, and protect resources. Resources are defined as personal attributes, conditions, objects, or energies that are valuable to an individual or that serve as a means for obtaining these factors (15). The potential or actual loss of resources is inherently threatening and can be a source of stress for individuals. Individuals use many strategies to deal with stressful situations or possible resource loss. These include developing and investing in resource surpluses to offset future resource loss, replacing the lost resources directly, symbolically, or indirectly with other resources, or reevaluating the value of lost resources to lessen their impact. Unfortunately, these approaches for dealing with resource loss cannot be applied to sleep because sleep has no alternative. We predict that individuals with insufficient sleep can become vulnerable to additional resource loss in what COR theory describes as a “loss spiral” (13, 14). Thus, this research aimed to answer an important question: can the loss of sleep be stressful enough to trigger the depletion of additional resources? Figure 1 depicts the conceptual model.

Although the extant literature has examined the link between sleep and multiple attitudinal and performance outcomes, including burnout, job satisfaction, safety compliance, and workplace-related health (16–19) the mediating mechanisms that account for these relationships have not received equal attention. Therefore, we examined the association between insufficient sleep and employee burnout. Subsequently, we proposed psychological capital (PsyCap), a psychological resource as a possible mediating factor. PsyCap describes an individual’s psychological state of development and integrates four positive psychological resources: efficacy, optimism, hope, and resiliency (20). Previous research suggests that a person’s PsyCap is susceptible to change through external influence (21). We also explored the link between insufficient sleep and job satisfaction. Scott and Judge (22) found that employees who reported having a poor night’s sleep also indicated low job satisfaction. Particularly, they found this relationship to be partially mediated by emotions specifically hostility, joviality, and attentiveness. While their research revealed the affective mediators in the relationship between sleep and job satisfaction, little is known about other psychological processes or mediators that may explain this link. We addressed this literature gap by using a serial mediation model to examine the relationship between sleep and job satisfaction.

In sum, this study contributes to the literature by uncovering the mediating mechanisms that explain the insufficient sleep-burnout link and the insufficient sleep-job satisfaction link. In doing so, our study responds to calls for researchers to further explore the processes underlying the sleep-work outcome relationships (23). Using survey data and a serial mediation research model, these authors concluded that although sleep is a non-work factor, it has a spill-over effect from home to work.

## 2. Theoretical background and hypotheses

### 2.1. Insufficient sleep and burnout

When employees do not get sufficient sleep, the consequences are felt in the workplace. Indeed, Ghumman and Barnes (24) found that when employees have a poor night of sleep, they lack the self-control necessary to restrict the explicit expression of their stereotypes and prejudice. Additionally, burnout is theorized an outcome of poor sleep (19). Burnout is defined as a psychological syndrome in response to interpersonal stressors (25). It encompasses three main dimensions: emotional exhaustion, depersonalization or detachment from the job, and a reduced sense of personal accomplishment (25). Emotional exhaustion refers to fatigue resulting from the depletion of one’s physical and emotional resources. Depersonalization reflects an indifferent or detached attitude toward work, while a reduced sense of personal accomplishment represents a lack of self-efficacy and achievement about social and non-social job accomplishments (26). However, it has been argued that reduced self-efficacy is not a core element of burnout but may rather be a consequence of burnout instead (27). Following this trend, our study focused on emotional exhaustion and depersonalization when referring to burnout.

Actual or even potential resource loss is powerful enough to cause psychological distress (including burnout) (28). Hobfoll (29) presented a comprehensive set of 74 resources validated in many Western contexts. They included the following: status, hope, optimism, stable employment, adequate income, and time for adequate sleep among others. The resource investment principle of the COR theory asserts that individuals must invest in resources as a buffer against the possibility of resource loss, to recover from loss, and to gain further resources (14). Unfortunately, unlike most resources, sleep is not a resource that can be replaced or compensated for with other resources. Thus, when an individual loses sleep, the potential consequences become especially significant. Similarly, Toker and Biron (30) explained that ineffective coping behavior to replenish resources may lead to further depletion of resources.

We theorize that employees with insufficient sleep are likely to experience burnout, specifically, emotional exhaustion and disengagement at work. Indeed, sleep complaints and indications of impaired sleep have been reported by individuals who experience burnout (31). Previous research suggests that poor sleep impacts emotional fatigue (22). When employees are sleepy, they are less cooperative, helpful, and courteous (towards others) because their ability to use information and discern emotions declines (12). Following previous assertions that sleep may be an indicator of employee stress levels (32), and empirical studies showing sleep as a predictor of burnout (19, 33), we posit the following:

*H1*: insufficient sleep will be related to burnout, such that employees with insufficient sleep will experience higher burnout compared to those with sufficient sleep.

### 2.2. The mediating role of psychological capital

Burnout has been described as a response to stress. Thus, it is likely that PsyCap may be a mediating mechanism that explains the association between sleep and burnout. PsyCap is a higher-order construct that describes an individual’s psychological state of development and integrates four positive psychological resources: (1) having the confidence to successfully tackle challenging tasks (efficacy), (2) maintaining a positive outlook about current and future success (optimism), (3) persevering towards the attainment of goals, and redirecting paths when required for success (hope), and (4) manifesting the ability to sustain and bounce back even stronger to achieve success in the face of adversity (resiliency) (20). PsyCap is a critical resource that employees use to cope with or minimize the symptoms of stress (32). PsyCap is referred to as a state-like psychological resource in that it is susceptible to external influence and development. In fact, a distinguishing characteristic of PsyCap is its malleability and ability to change over time (21). Emerging sleep literature has found that poor sleep may contribute to the depletion of a person’s psychological resources. For example, Barnes and colleagues (22) asserted that because an individual’s self-regulatory resources are depleted daily, and replenished during sleep, poor sleep results in ego depletion.

Extant literature suggests a relationship between sleep and the four positive psychological resources (i.e., hope, efficacy, resilience, and optimism) that make up the construct. For instance, Barnes (34) reported that sleep is necessary to be able to direct and focus attention on specific tasks. Thus, insufficient sleep may have an impact on the agency and determination to achieve goals that the motivational state of hope engenders. Additionally, prior research found a correlation between sleep and self-efficacy (35). Specifically, the researchers found that study participants with sleep problems reported lower self-efficacy than individuals without such problems. Similarly, it has been suggested that unhealthy sleep habits could hinder resilience to both current and future adversities and stressors (36). Moreover, results of a lab experiment by Haack and Mullington (37) concluded that insufficient sleep may cause a decline in psychosocial functioning and positive outlook, ultimately affecting optimism. Empirically, sleep has been linked to PsyCap (38).

Research suggests that individuals with high PsyCap possess extra resources that enable them to mentally reframe and reinterpret situations more positively (39), so that when challenged with setbacks and obstacles, they can persevere and not give up. Following the COR theory, which suggests that people employ valued resources to respond to stress and to build a reservoir for future needs, the stress literature has identified PsyCap as a mediator between stressful contexts and burnout. PsyCap has been found to be a mediator between occupational stress and burnout (40), between workaholism and burnout (41), and between workplace bullying and burnout (42). Following theoretical and empirical literature, we hypothesized that insufficient sleep would positively influence burnout through PsyCap.

*H2*: psychological capital will mediate the relationship between insufficient sleep and burnout, such that insufficient sleep depletes psychological capital, and the decrease of psychological capital leads to higher burnout.

### 2.3. Insufficient sleep and job satisfaction

Locke (43) offered one definition of job satisfaction as “…a pleasurable or positive emotional state resulting from the appraisal of one’s job or job experiences” (p. 1304). Saari and Judge (44) contended that perceptions of emotion and cognition are implicit in Locke’s definition of job satisfaction. That is, people’s thoughts and feelings factor into the overall evaluation of their jobs. Lab experiments demonstrate that sleep loss results in glucose hypometabolism (45), leading to a decrease in the deactivation of the whole brain particularly in the prefrontal cortices, causing deficits in cognitive functioning (12). Scott and Judge (22) found that poor quality of sleep accounted for within-individual variances in multiple emotions, including hostility and joviality. Other researchers have also shown that poor quality of sleep impairs the ability to recognize and properly process emotions (46).

When individuals experience resource loss, their vulnerability to further resource loss increases (13). Ritter and colleagues (47) suggested that job satisfaction is an affective resource, which can influence a person’s experience of future stressors. They argue, for instance, that “the accumulation of a resource, such as job satisfaction, may enhance one’s ability to seek and perceive role clarity, such that subsequent assessments of role clarity will be higher after experiencing high levels of job satisfaction” (p. 1658). Therefore, it is quite likely that employees who report insufficient sleep will also report lower job satisfaction. Consistent with this argument, a study found a positive association between sleep and job satisfaction, and satisfaction subsequently mediated the relationship between sleep and organizational citizenship behavior directed toward both peers and the organization (16).

*H3*: insufficient sleep will be related to job satisfaction, such that employees with insufficient sleep will experience lower job satisfaction compared to those with sufficient sleep.

### 2.4. Sequential mediating role of psychological capital and burnout between insufficient sleep and job satisfaction

As noted previously, individuals often try to employ other resources to offset the net loss. Losing resources is stressful. Thus, when an individual lacks the resources to offset resource loss, “loss spirals” may ensue because people become more vulnerable to additional resource loss (13, 14). This concept aligns with the resource caravan principle of the COR theory, which posits that resources do not exist in isolation but instead travel in caravans or packs (28). Hobfoll and colleagues (13) supported this by pointing to the high correlation often found among variables such as self-efficacy, self-esteem, and optimism, and explaining that these factors are likely to develop from a common supportive environment. In a similar vein, we expect that insufficient sleep would lead to the depletion of additional resources in the workplace. In a recent review (9), several studies demonstrated that poor sleep had multiple negative consequences in the workplace concerning employees’ health, cognition, emotions, attitudes, and behaviors. When employees do not meet their sleep needs, they experience a depletion of their psychological resources. The diminishing of psychological resources impairs the employees’ ability to effectively deal with work stress, thereby leading to burnout (48). The depletion of psychological resources along with the resultant burnout is likely to induce lower job satisfaction. Empirical evidence that supports this view includes research that found a significant relationship between sleep and PsyCap (38), between PsyCap and burnout (32), and between burnout and job satisfaction (49).

These findings together with the prior hypotheses suggest a serial mediation model or relationship. A serial mediation model describes a model with multiple mediators in which mediators causally affect each other or, more generally, the variables in the model affect each other in a causal sequence. Using a serial mediation model, prior research found that supervisors’ sleep quality depleted their ego, which then induced abusive supervision and finally impacted the unit’s work engagement (23). Specific to our study, we integrated all the variables in our model and predicted a serial relationship in which the relationship between sleep and job satisfaction is transmitted first through PsyCap, and subsequently, through burnout.

*H4*: psychological capital and burnout will serially mediate the relationship between insufficient sleep and job satisfaction, such that insufficient sleep decreases psychological capital, which in turn increases burnout, and the increased burnout reduces job satisfaction.

## 3. Methods

### 3.1. Sample and procedure

To test the study hypotheses, cross-sectional survey data were collected from employees of a call center, a stressful work environment that requires high service quality and customer satisfaction without compromising response time (50). Employees in call centers are required to display prescribed emotions that are not always congruent with the emotions they are experiencing. This emotional conflict or dissonance along with the lack of control associated with the work environment, makes burnout a psychological syndrome characteristic of call centers (51). We reached out to and obtained permission from the managers of a call center for a government-owned public bank situated in Seoul, South Korea. The center is comprised of multiple teams providing varied services. Approximately 44% of the sample comprised individuals in active roles who handle outbound calls and are responsible for marketing the bank’s financial products and services. 36% of them in more passive roles take care of inbound calls and provide general and professional consultation to clients. Ten percent of the sample were part of an administrative team that monitors call quality and provides training and education for employees as required. The rest is comprised of a management team that oversees the center. All employees were Korean nationals.

Although all the measures were originally developed in English, this study used the Korean language throughout the survey. Following recommendations by Brislin (52), we performed standard translation and back-translation for study variables to ensure measurement equivalence. Before data collection, management announced the research to employees. Employees were required to sign an informed consent form to participate. The survey was completed through the on-site administration of a paper-and-pencil questionnaire. Each participant was given a questionnaire package in a sealed envelope to ensure anonymity. The completed surveys were sealed and returned to a designated spot. Through cooperation with top management of the call center, 400 questionnaires were distributed to the employees, and all distributed surveys were returned to researchers directly. After data cleaning to delete the data of participants with missing values, a sample size of 322 remained. The data collection process is presented in Appendix A. The participants were predominantly female (89.75%), which is comparable to gender ratios in prior call center studies (53), and their ages ranged from 20 to over 50 years (please see Table 1).

**Table 1:** Demographic characteristics of sample.

Variable	Percent
Age (in years)	
20–24	2.17%
25–29	9.01%
30–34	17.08%
35–39	12.11%
40–44	16.15%
45–49	13.66%
>50	29.82%
Gender	
Female = 1	89.75%
Male = 2	10.25%
Organizational tenure (in years)	
<0.25	20.50%
1	19.25%
2–4	22.05%
5–7	13.98%
8–10	15.22%
11–13	4.35%
>14	4.65%
Position	
Employee	81.05%
Manager	18.95%

### 3.2. Measures

This study analyzed all the variables at the individual level. Unless otherwise indicated, the study variables were measured with a 7-point Likert scale (1 = strongly disagree, 7 = strongly agree). We also assessed internal reliabilities using Cronbach’s alpha values.

#### 3.2.1. Insufficient sleep

Subjective measures of sleep correlate well with objective measures of sleep (54) and are consistent with prior sleep studies (55, 56). Additionally, it has been argued that, unlike many variables in organizational research, a single-item measure for sleep proves beneficial for measuring sleep and has been found to be substantially correlated with health, affective, and attitudinal outcomes (9). Thus, we assessed participants’ sleep using a single-item: “I evaluate my sleep time as insufficient.”

#### 3.2.2. Psychological capital

Employees’ PsyCap was assessed using the 12-item PsyCap Questionnaire (PCQ) (57). The PCQ-12 comprises four sub-dimensions: self-efficacy (three items), hope (four items), resilience (three items), and optimism (two items). Sample items include, “I feel confident in representing my work area in meeting with management” and “I feel confident contributing to discussions about the company’s strategy” for efficacy, “If I should find myself in a jam at work, I could think of many ways to get out of it” and “At this time, I am meeting the work goals that I have set for myself” for hope, “I usually take stressful things at work in stride” and “I can get through difficult times at work because I’ve experienced difficulty before” for resilience and “I always look on the bright side of things regarding my job” and “I’m optimistic about what will happen to me in the future as it pertains to work” for optimism. The internal consistency reliability is 0.91.

#### 3.2.3. Burnout

The seven items of the burnout scale from Kalliath and colleagues (58) were used to measure burnout. The scale contains two sub-dimensions: emotional exhaustion (five items) and depersonalization (two items). Sample items include, “I feel frustrated by my job,” and “I feel fatigued when I get up in the morning and have to face another day on the job” for emotional exhaustion, and “I worry that this job is hardening me emotionally” and “I’ve become more callous towards people since I took this job” for depersonalization. The internal consistency reliability is 0.91.

#### 3.2.4. Job satisfaction

Three items were used to measure job satisfaction (59). Sample items are “Generally speaking, I am satisfied with this job” and “I frequently think of quitting this job (Reverse-coded). The internal consistency reliability is 0.92.

#### 3.2.5. Control variables

Research suggested that gender and age have an impact on individual sleep needs (60). Thus, following previous research (61, 62), we controlled for demographic variables including age, gender, tenure, and position to parcel out their effects on the dependent variable, job satisfaction. Age was measured in intervals and coded as 1 for 20–24 years up to 7 for more than 50. Tenure was also coded as 1 for less than three months up to 7 for more than 14 years. Gender was dummy coded as 1 for female and 2 for male with position similarly coded as 1 for employee and 2 for manager (please see Table 1 for details).

### 3.3. Statistical analyses

Statistical analyses were performed using STATA 14.1 (63). We first calculated Cronbach’s alpha to assess the internal reliability of the measures. The mean scores of the demographic variables, sleep, PsyCap, burnout, and job satisfaction, were computed for the Pearson correlational analyses. Because of the multiple measurement items in the hypothesized model, we used item parceling (64). Generally, parceling allows for fewer parameter estimates, the reduced sampling error for better estimates, a lower indicator-to-sample size ratio, and a lower likelihood of correlated residuals and dual-factor loadings (64). Specifically, the multidimensional constructs were parceled by averaging the individual items of each dimension to create four and two parceled indicators for PsyCap and burnout, respectively. We conducted Confirmatory Factor Analysis (CFA) to assess the construct validity of our measures. The comparative fit index (CFI), root mean square error of approximation (RMSEA), and Tucker-Lewis Index (TLI) were used to evaluate the goodness of fit of the hypothesized research model using the stringent cut-off points of 0.95, 0.05, and 0.95, respectively. We set alternative models and performed the chi-square difference test to further verify the model. The direct effect hypotheses (H1 and H3) were tested with regression analyses using robust standard errors to mitigate concerns of non-normality of the data distribution (65). We conducted bootstrapping analysis using 10,000 bootstrapped samples, to test the indirect effect of the mediation (H2) and serial mediation (H4) hypotheses. We use *p* < 0.05 as the cut-off for model and path co-efficient significance tests.

## 4. Results

### 4.1. Model validity and common method bias checks

CFA was performed to examine the factor structure of the multi-item study variables. First, we estimated the hypothesized three-factor model. As presented in Table 2, the goodness of fit indicators met the cut-off points: *χ*^2^ = 38.54; df = 20, *p* < 0.01; *χ*^2^/df = 1.93; CFI = 0.99; TLI = 0.98; RMSEA = 0.05 (66). The factor loadings for the variables of interest were above the 0.50 cut-off value (67) ranging from 0.63 to 0.89 at a statistically significant level (*p* < 0.001). Four alternative models were also tested. In the first alternative model, where PsyCap and job satisfaction were loaded on the same factor, the model fit the data significantly worse, CFI = 0.93; TLI = 0.88; RMSEA = 0.13. The fourth alternative model with all variables loaded on one also performed significantly poorly, CFI = 0.88; TLI = 0.81; RMSEA = 0.17. In sum, the results revealed that the hypothesized model is distinct from the alternative models and presents the best-fit indicators. To check convergent validity, average variance extracted (AVE) and composite reliability (CR) were calculated for each construct, and they satisfied the standard of being above 0.50 and 0.70, respectively (67). Further, the inter-construct correlation values were lower than the square root of the AVE, satisfying the criterion for discriminant validity (67).

**Table 2:** Model fit statistics for measurement models.

Measurement model	*χ* ^2^	df	CFI	TLI	RMSEA	Δ*χ*^2^	Δdf
Baseline (hypothesized) three factor model	38.54**	20	0.99	0.98	0.05		
Alternative 1 (two factor model)^a^	148.14***	22	0.93	0.88	0.13	109.60***	2
Alternative 2 (two factor model)^b^	170.70***	22	0.91	0.86	0.14	132.16***	2
Alternative 3 (two factor model)^c^	121.20***	22	0.94	0.91	0.12	82.66***	2
Alternative 4 (one factor model)^d^	237.69***	23	0.88	0.81	0.17	199.15***	3

a Two-factor model with psychological capital and job satisfaction on the same factor.

b Two-factor model with psychological capital and burnout on the same factor.

c Two-factor model with burnout and job satisfaction on the same factor.

d One-factor model with psychological capital, burnout, and job satisfaction on the same factor.

*N* = 322. **p < 0.01 and ***p < 0.001 (two-tailed test).

Due to the cross-sectional nature of the data, common method bias (CMB) may be of concern. Thus, we performed Harman’s one-factor analysis to rule out the possibility that the variance in the data largely stems from one factor. The results show that no single factor accounted for more than 29% of the covariance among the variables suggesting that CMB may not be a pervasive issue (68).

### 4.2. Descriptive statistics

The descriptive statistics and inter-correlations results are presented in Table 3. As expected, insufficient sleep correlated negatively with psychological capital (*r* = −0.20, *p* < 0.001) and negatively with job satisfaction (*r* = −0.29, *p* < 0.001). Psychological capital correlated negatively with burnout (*r* = −0.27, *p* < 0.001) and positively with job satisfaction (*r* = 0.60, *p* < 0.001). After conducting the regression analyses, the variance inflation factors of variables in the estimations were all below 10. Thus, we determined multicollinearity would not be high (69).

**Table 3:** Descriptive statistics and inter-correlations.

	Mean	SD	1	2	3	4	5	6	7	8
1. Age	4.91	1.82	–							
2. Gender	1.10	0.30	−0.29***	–						
3. Tenure	1.72	0.49	0.23***	−0.10	–					
4. Position	2.10	0.99	−0.22***	0.15**	0.23***	–				
5. Insufficient Sleep	3.30	1.48	−0.20***	0.05	−0.12*	0.06	–			
6. Psychological Capital	4.60	0.80	−0.05	0.03	0.07	0.01	−0.20***	(0.91)		
7. Burnout	4.05	1.15	−0.16**	0.07	−0.01	0.23***	0.25***	−0.27***	(0.91)	
8. Job satisfaction	4.41	1.26	0.04	−0.07	0.16**	0.08	−0.29***	0.60***	−0.46***	(0.92)

*N* = 322. Cronbach’s alpha values are reported in parentheses on the diagonals. *p < 0.05, **p < 0.01, and ***p < 0.001 (two-tailed).

### 4.3. Hypotheses testing

#### 4.3.1. Hypotheses testing of the direct effects

The results of the ordinary least squares regression analyses are presented in Table 4. Hypothesis 1 posited that employees who report insufficient sleep are more likely to experience burnout compared to those with sufficient sleep. Consistent with our prediction, the regression results show a significant positive relationship between insufficient sleep and burnout (*b* = 0.13 (0.04), *p* < 0.01, Model 2). Hypothesis 3 posited that employees with insufficient sleep are more likely to experience lower job satisfaction compared to those with sufficient sleep. The results provide support for the hypothesis (*b* = −0.09 (0.03), *p* < 0.01, Model 3).

**Table 4:** Results of regression analyses and bootstrapped indirect effect test.

Main effects	Psychological capital (Model 1)	Burnout (Model 2)	Job satisfaction (Model 3)
Age	−0.04 (0.03)	−0.05 (0.04)	−0.01 (0.03)
Gender	0.02 (0.15)	0.04 (0.18)	−0.29 (0.17)
Tenure	0.02 (0.03)	0.00 (0.04)	0.06* (0.03)
Job level	0.05 (0.07)	0.34*** (0.08)	0.21* (0.08)
Insufficient sleep (IS)	−0.12** (0.03)	0.13** (0.04)	−0.09** (0.03)
Psychological capital (PC)		−0.36*** (0.09)	0.76*** (0.07)
Burnout (BO)			−0.37*** (0.06)
Job satisfaction (JS)			
*F*	3.70**	10.96***	53.10***
*R* ^2^	0.06	0.17	0.50
Δ*R*^2^		0.11	0.33

Indirect effects	Estimate	Lower level	Upper level
IS → PC → BO	0.04	0.02	0.08
PC → BO → JS	0.16	0.09	0.25
IS → PC → BO → JS	−0.02	−0.03	−0.01

*N* = 322. Estimates for regression were calculated using robust standard errors reported in parentheses. The indirect effects estimate was tested for significance using 95% bias-corrected bootstrapped confidence intervals (10,000 times). *p < 0.05, **p < 0.01 and ***p < 0.001 (two-tailed).

#### 4.3.2. Hypotheses testing of the indirect effects

Hypothesis 2 posited that PsyCap will mediate the relationship between insufficient sleep and burnout. To test this simple mediation, we performed a bootstrapping analysis using 10,000 bootstrapped samples. We utilized the 95% bias-corrected confidence interval (CI) for the indirect effect. The mediation coefficient was 0.04 with a CI that did not include zero [0.02, 0.08]. The final hypothesis predicted a serial mediation wherein insufficient sleep is related to job satisfaction via PsyCap and burnout sequentially. The indirect test with 10,000 bootstrapped samples produced a coefficient of-0.02 and the 95% CI also excluded zero [−0.03, −0.01]. Altogether, these results provide support for hypotheses 2 and 4.

## 5. Discussion

Sleep is an important non-work factor that affects work. Using a cross-sectional design, we investigated the relationship between sleep and workplace outcomes. The results supported all the study hypotheses. The theoretical and practical implications of the study results are discussed below.

### 5.1. Theoretical implications

To begin with, our study focused on individual subjective beliefs about meeting one’s sleep needs. This choice aligns with the literature that suggests that the optimal sleep needs of each person vary by medical, genetic, behavioral, and environmental factors (70). Particularly, operationalization of sleep (9) has often centered around the number of hours slept, staying asleep, struggling to fall asleep, the number of mid-sleep awakenings throughout the night, or whether a person feels rested after waking up. Following recommendations by Litwiller and colleagues (9) about the benefits of a single-item measure of sleep, our use of insufficient sleep, measured by individual perception of the inability to meet one’s sleep needs contributes to the sleep literature.

Most research on sleep within the organizational sciences has focused on the main effects of sleep (34). In recent meta-analytic research on the relationship between sleep and work (9), the researchers noted that due to the lack of primary data, it was impossible to test the mediating processes that explain the main effects of sleep. Of the extant studies that examine the role of mediators, a large majority have focused on information processing, emotion, and affect variables (12, 22) with a few on cognitive mediators (17). This research extends the sleep literature by investigating the explanatory mechanisms of sleep from a cognitive and attitudinal perspective. We find that PsyCap and burnout are processes that underlie some of the main effects of sleep and work.

Previous studies that examine the impact of sleep on burnout have focused largely on the direct effect as well as the bi-directional relationship between these two variables (19, 33). Using a cross-sectional dataset from a South Korean bank call center, the current study corroborated research that reported a positive relationship between insufficient sleep and burnout. In addition, the study presented and found support for PsyCap as a mediating variable that explains the relationship between these variables. While we did not present a hypothesis for the direct effect of sleep on PsyCap, the regression results support this link. This finding supports early empirical work (38) linking sleep to psychological capital.

This study further deepens our understanding of how sleep impacts job satisfaction. Prior research by Scott and Judge (22) suggested that the emotions of hostility, joviality, and attentiveness mediate the relationship between sleep and job satisfaction. The resource loss and resource caravan principle of the COR theory is used to unravel other mediating mechanisms that may contribute to this relationship. We posited that insufficient sleep may trigger additional losses and adopted a serial mediation model to explain the insufficient sleep-job satisfaction link. Specifically, our findings suggest that insufficient sleep is associated with the loss of further resources leading to the depletion of PsyCap. The decreased PsyCap increases employee burnout and finally, the increased burnout reduces job satisfaction. Overall, the significant effects of sleep loss on multiple workplace outcomes underscores the spill-over effects of sleep (16).

### 5.2. Practical implications

The study’s findings make several contributions to practice. First, our results suggest that when employees are unable to adequately meet their sleep needs, it increases the likelihood of burnout and reduces job satisfaction. These findings align with prior studies that found that poor sleep impacts employees’ mental state and attitudes at work (16, 24, 37). Thus, organizations need to cultivate a healthy sleep culture among employees (71). This can be achieved through education and training campaigns that encourage employees to prioritize their sleep needs. Employees should be introduced to the scientific evidence on sleep and its effects on employee well-being, performance, and productivity. Negative notions that glorify sleep as disposable and antithetical to success need to be dispelled. Through a top-down approach, managers and supervisors must be encouraged to model healthy sleep behaviors that trickle down to employees.

For call center managers, this would require investing in optimal scheduling processes and technologies (50) that are mindful of employees’ sleep needs. This ensures that agents are not overstrained by unsustainable work shifts that are detrimental to their sleep patterns but also that the agents are well-rested and in the right frame of mind to deliver quality customer service to clients. Additionally, incorporating flexible scheduling that allows agents to change shifts or self-schedule within reason would be instrumental in minimizing burnout and improving job satisfaction in an industry notorious for high employee attrition and turnover rates.

Second, the advancement of communication technology has improved connectivity, providing flexibility to work from any place without being limited by proximity (72). On the other hand, technology has blurred the boundary between working and non-working hours and an increasing number of employees are bringing work home by responding to emails, texts, and other work correspondence. This issue was compounded by the COVID-19 pandemic as the fraction of the workforce working remotely increased significantly. While remote work has reduced commute time and should translate to more time for sleep, maintaining boundaries between professional and personal lives can be difficult with employees reporting more work hours as a result (73). Thus, organizations need to have clearly delineated boundaries by instituting stricter “off hours” and limiting communication at these times (23).

Third, managers may need to pay particular attention to the sleep needs of employees in the service sector. The attitude and behavior of these employees significantly influence customer perceptions of service quality (74). Additionally, dealing with customers can be quite stressful as employees are expected to exhibit emotional intelligence and maintain a positive attitude while addressing issues in a timely manner. Thus, managers must ensure that these employees are not overtasked with extended work hours that may significantly alter their sleep (71).

Fourth, the study showed that the influence of insufficient sleep on employee burnout operates via PsyCap. PsyCap is an important psychological resource with empirical research demonstrating its importance within the work context (40). In fact, many organizations when hiring look for employees with high PsyCap while others conduct training interventions and programs to develop the PsyCap of their employees (21). While these are important, our study suggests that sleep is another factor that managers should consider because poor sleep may have a negative impact on an individual’s PsyCap.

### 5.3. Limitations and directions for future research

Although our research possesses several theoretical and practical implications for sleep research, it also has some limitations. For one, the study uses cross-sectional dataset. When the same participant responds to both the predictor and outcome variables, self-report bias may ensue and result in inflated covariance between the variables. Although the results of the Harman’s one-factor analysis show that no single factor accounted for more than 29% of the covariance among the variables, there should be cautious interpretation of the results. The use of cross-sectional data also means that causal inferences cannot be established between the study variables.

As presented, this study starts from insufficient sleep and flows towards PsyCap, burnout, and job satisfaction. However, it is important to note that organizational and occupational stressors are predictors of poor sleep (9), suggesting that the predicted directional relationships may stem from stress. The possibility of reverse causality may exist among some of the variables. For instance, PsyCap may not only be an outcome of insufficient sleep but may also predict sleep. Future studies should conduct time-lagged and longitudinal research and employ network analysis to untangle the directional relationships. Experimental designs are also useful to establish causality between variables and to alleviate problems of endogeneity in research models (75). Thus, there is a need for experiment designed to examine the relationship between sleep and other work outcomes.

The distribution of variables including gender, organizational tenure, and position did not follow a normal distribution which is the reality in the field. For one, employees in the call center industry tend to be predominantly female (53) with a high turnover rate. In addition, the age distribution is reflective of the rapidly aging workforce in South Korea where older adults (50–64 years) make up about 37.5% of the working-age population (76). While we mitigated concerns of non-normality of the data distribution by conducting the regression analysis using robust standard errors, as they are less dependent on the normality assumption (65), non-normality of the data remains a limitation of the study.

Our measurement of sleep is static and does not capture the within-person variations in sleep. However, choices are made daily in allocating time towards sleep versus other activities such as work and family (77). Research must follow the current trend that uses the daily approach such as the diary studies or experience sampling method research design (23). The sleep variable was also measured with a single-item instrument which critics say is inadequate to capture complex concepts and for which internal reliability cannot be calculated (78). However, unlike many other topics, a single-item measure of sleep has been found to be substantially correlated to workplace outcomes, and thus much of the benefit of measuring sleep can be gained from such measures (9). In fact, several sleep studies use single item measure (56). Nonetheless, multi-item and objective measures of sleep may help to address some of the inconsistencies in the relationship of the sleep-work outcome.

There is also the need to consider multilevel research designs as well as potential boundary conditions of the relationships in the study. Individual differences or work environments may serve as potential moderators. Future research could also extend the sleep literature by examining the effects on other work outcomes and paying attention to the mediating mechanisms that explain such relationships.

## 6. Conclusion

While sleep did not play a significant role in traditional management or human resource literature (79, 80), in recent times, organizational scholars have been paying increasing attention to sleep. This research contributes to the extant literature by investigating the effects of insufficient sleep on workplace outcomes. Insufficient sleep was associated with burnout. This link was found to have been mediated by PsyCap. Insufficient sleep was also associated with job satisfaction, and this link was serially mediated first by PsyCap, followed by burnout. Using the COR theory, our findings suggest that as an irreplaceable resource, losing sleep may trigger additional loss of resources important for work. Despite the limitations, our research contributes to advancing the sleep literature.

## Data availability statement

The raw data supporting the conclusions of this article will be made available by the authors, without undue reservation.

## Author contributions

MO was the principal researcher and prepared the first draft of the article under the supervision of S-WK. SC added valuable theoretical and methodological insights based on his knowledge and expertise regarding the topic of this study. S-WK supervised the study and refined the draft into a publishable article. All authors contributed to the article and approved the submitted version.

## Funding

This work was supported by the Ministry of Education of the Republic of Korea and the National Research Foundation of Korea (NRF-2022S1A5A2A01048556).

## Conflict of interest

The authors declare that the research was conducted in the absence of any commercial or financial relationships that could be construed as a potential conflict of interest.

## Publisher’s note

All claims expressed in this article are solely those of the authors and do not necessarily represent those of their affiliated organizations, or those of the publisher, the editors and the reviewers. Any product that may be evaluated in this article, or claim that may be made by its manufacturer, is not guaranteed or endorsed by the publisher.

## Figures and Tables

**Figure A1:**
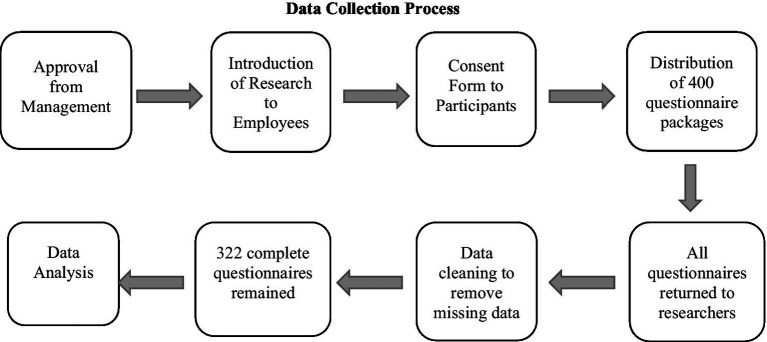
Data collection process.
